# Choice Food Pantries in the Deep South: A Novel Approach to Addressing Food Insecurity in Older Adults

**DOI:** 10.1093/geroni/igaa057.2795

**Published:** 2020-12-16

**Authors:** David Buys, Masey Smith, Erin King

**Affiliations:** Mississippi State University, Mississippi State, Mississippi, United States

## Abstract

Older adults in the deep south are living with high food insecurity rates; this is exacerbated by challenges with rural-living, like transportation limitations and no grocery stores. To address this, we must increase emergency food assistance offerings and adopt best practices for food pantries including choice food pantry approaches, which empowers clients with some autonomy in choosing the foods they receive as part of their pantry distribution. Coalitions in eight income-limited, aging, rural Mississippi Delta counties received support from a Centers for Disease Control and Prevention Grant to enhance the food-related infrastructure in their communities through technical assistance and economic investments. A detailed process evaluation was conducted on this effort. Each coalition adopted food pantry-related policies like adding new food pantries and adapting their existing food pantries with the choice model. Both aging volunteers and clients indicated positive outcomes from the process of adding pantries and adapting existing ones.

